# An analysis of HER2 amplification in cervical adenocarcinoma: correlation with clinical outcomes and the International Endocervical Adenocarcinoma Criteria and Classification

**DOI:** 10.1002/cjp2.184

**Published:** 2020-10-22

**Authors:** Haiyan Shi, Ying Shao, Weiguo Lu, Bingjian Lu

**Affiliations:** ^1^ Department of Surgical Pathology, Women's Hospital School of Medicine, Zhejiang University Hangzhou PR China; ^2^ Center for Uterine Cancer Diagnosis and Therapy Research of Zhejiang Province Women's Hospital, Zhejiang University Hangzhou PR China; ^3^ Department of Gynecological Oncology, Women's Hospital School of Medicine, Zhejiang University Hangzhou PR China

**Keywords:** cervical adenocarcinoma, gastric‐type, HER2, gene amplification, HPV, prognosis

## Abstract

Few studies have explored HER2 status in cervical adenocarcinoma, particularly in the gastric‐type adenocarcinoma (GAC), a nonhuman‐papillomavirus‐related subtype with poor clinical outcomes. In this study, we investigated HER2 expression and amplification by immunohistochemistry (IHC) and fluorescence *in situ* hybridization (FISH) in 209 well annotated cervical adenocarcinomas diagnosed using the International Endocervical Adenocarcinoma Criteria and Classification. IHC identified HER2 protein expression in 57.4% (123/209) of adenocarcinomas, of which 62 were IHC 1+ (negative), 38 2+ (equivocal) and 23 3+ (positive). *HER2* amplification was found in 13 cases (6.2%) including 10 with IHC 3+ and 3 with IHC 2+. Among all the major histotypes of cervical adenocarcinoma, *HER2* amplification was most common in GAC cases with a frequency of 14.7% (5/34). Moreover, *HER2* amplification was more frequently associated with 2018 International Federation of Gynecology & Obstetrics (FIGO) stage III/IV, perineural involvement and ovarian spread (*p* < 0.05) while IHC 3+ was more common in patients with lymphovascular invasion and ovarian involvement (*p* < 0.05). Survival analysis indicated that FIGO stage III/IV, GAC, and p53 overexpression were associated with poor disease‐specific survival and tumor recurrence (*p* < 0.05). In conclusion, *HER2* amplification was present in a subset of adenocarcinomas, and more common in GAC, pointing to a potential benefit from trastuzumab treatment. HER2 overexpression does not identify gene amplification status in cervical adenocarcinoma; therefore, FISH is suggested for both IHC positive and equivocal cases. Further investigation on more cases with longer follow‐up times is required to consolidate these findings.

## Introduction

Cervical carcinoma is the fourth most frequent cancer worldwide in women, with an estimated 570 000 new cases in 2018 [1]. Adenocarcinoma is the second most common histotype, accounting for 10–25% of all cervical carcinomas [2], and both the relative incidence and absolute number of endocervical adenocarcinomas have been increasing over the last two or three decades. Compared with squamous cell carcinoma, cervical adenocarcinoma has been found to have a poorer prognosis although there are no differences in the treatment strategy between them [3, 4]. Moreover, cervical adenocarcinomas are a heterogeneous group of tumors that may harbor prognostic differences. An international group of gynecological pathologists has recently proposed a new pathogenetic scheme, the International Endocervical Adenocarcinoma Criteria and Classification (IECC), which is based on the presence or absence of high‐risk (HR) human papillomavirus (HPV) infection‐related morphological features [5]. The IECC approach appears to be valuable for prognostic assessment [6, 7]. In particular, gastric‐type adenocarcinoma (GAC), the predominant type of non‐HPV‐related adenocarcinoma, has demonstrated poorer clinical outcomes compared with other histotypes of cervical adenocarcinoma [8, 9, 10, 11, 12]. It is a pressing requirement to identify biomarkers that have potential therapeutic significance to improve the prognosis in cervical adenocarcinomas.

Our knowledge of human epidermal growth factor receptor 2 (*HER2*) amplification has expanded from breast carcinoma to other human carcinomas including gastroesophageal cancer and uterine serous carcinoma because HER2‐positive cancers may benefit from anti‐HER2 monoclonal antibody therapy (trastuzumab) [13, 14, 15]. Several studies have investigated HER2 expression by immunohistochemistry (IHC) in cervical carcinomas including a small number of adenocarcinomas [16, 17, 18]. However, some important relevant aspects remain unresolved in cervical adenocarcinomas, such as the clinical significance, the precise prevalence and the histotype distribution of HER2 overexpression, and the concordance between HER2 immunohistochemical results and gene amplification. To address these issues, we performed a systematic analysis of HER2 expression and gene amplification in a large cohort of cervical adenocarcinomas.

## Materials and methods

### Patients and pathological review

The hospital's Institutional Review Board approved this study (IRB: 20170139; October 2017). In total, 209 adenocarcinomas were retrieved from the archives of the Department of Surgical Pathology, Women's Hospital, School of Medicine, Zhejiang University, PR China, between January 2014 and June 2019. The clinical and demographic features of the cases are given in supplementary material, Table S1. Rare subtypes including the serous type and the endometrioid type were excluded from the present study if they had concurrent carcinomas in the uterus, the Fallopian tube and/or the ovary. The clinical data were extracted from the electronic medical records before de‐identification. Tumor stage was re‐assessed according to the 2018 International Federation of Gynecology & Obstetrics (FIGO) cervical cancer staging system [19]. Two authors (LB and SH) independently reviewed the archival H&E slides for detailed morphological analysis. All cases were reclassified according to the IECC [5]. The pattern of invasion in endocervical adenocarcinomas, usual‐type, was determined according to a three‐tier pattern‐based histopathologic system [20]. The patients were followed up by chart review and telephone communication until June 2020.

### Immunohistochemistry

IHC was carried out on 3‐μm thick sections from neutral‐buffered formalin‐fixed, paraffin‐embedded tissue blocks. The antibodies included HER2 (4B5; Thermo Fisher Scientific, Waltham, MA, USA; ready‐to‐use), Ki67 (MIB1; Thermo Fisher Scientific; dilution 1:400), p16 (G175‐405; BD Bioscience, San Jose, CA, USA; 1:100), MUC6 (MRQ‐20; Cell Marque, Rocklin, USA; ready‐to‐use), p53 (DO‐7; Thermo Fisher Scientific; 1:300), estrogen receptor (ER) (SP1; Thermo Fisher Scientific; 1:300), and progesterone receptor (PR) (SP2; Thermo Fisher Scientific; 1:500). An En Vision immunostaining procedure (DAKO, Carpentaria, CA, USA) was performed according to the manufacturer's protocols. The antibodies were assessed by the percentage of positive cells (negative staining, <5%; 1+, 5–24%; 2+, 25–49%; 3+, ≥50%). Strong, diffuse nuclear staining (>70% cells with deep staining) was defined as p53 overexpression. HER2 staining was interpreted as 0 (negative), 1+ (negative), 2+ (equivocal), and 3+ (positive) following the criteria for scoring HER2 expression in gastric and gastroesophageal adenocarcinomas [21]. Two patterns of p16 immunoreactivity were identified – widespread, diffuse staining (block staining) and patchy positivity. Ki67 labeling index was calculated by counting in 10 high‐power fields from hotspots.

### HPV RNA *in situ* hybridization

HPV testing was performed by RNA *in situ* hybridization (ISH) with the RNAscope HPV‐HR18 Probe (Advanced Cell Diagnostics, Hayward, CA, USA), which recognizes 18 HR‐HPV genotypes (16, 18, 26, 31, 33, 35, 39, 45, 51, 52, 53, 56, 58, 59, 66, 68, 73, and 82) according to the manufacturer's instructions. The presence of a punctate yellow‐to‐brownish nuclear reaction was defined as positive staining for HPV RNA [22].

### Fluorescence *in situ* hybridization

A dual‐probe fluorescence *in situ* hybridization (FISH) assay for *HER2* and chromosome enumeration probe 17 (*CEP17*) was carried out using a PathVysion *HER2* DNA probe kit (Abbott Molecular Inc., Des Plaines, IL, USA) according to the manufacturer's protocol. Tumor nuclei were counterstained with 4′,6‐diamidino‐2‐phenylindole (DAPI) (Abbott Molecular Inc., Des Plaines, IL, USA) after hybridization. The *HER2* and *CEP17* signals per nucleus were recorded. The results from 20 interphase nuclei from invasive carcinomatous cells were reported as the ratio of total *HER2* signals to total *CEP17* signals. *HER2* testing was eventually interpreted as amplification or no‐amplification according to the updated American Society of Clinical Oncology/College of American Pathologists (ASCO/CAP) guideline for breast carcinoma (2018) [23].

### Statistical methods

The SPSS 16.0 software package (SPSS Inc., Chicago, IL, USA) was used for statistical analysis. Nonparametric tests (Wilcoxon test or Mann–Whitney *U* test) were used to determine the clinicopathological significance of HER2 expression/amplification. The Kaplan–Meier method was performed to compare mortality and recurrence between groups, and the log‐rank test was used to analyze the statistical significance of the differences between groups. Multivariate survival analysis was carried out by the Cox's proportional regression hazard (HR) method (conditional forward). We adjusted multiple parameters that were found to be significant in the univariate model. The statistical threshold was set at 0.05 (two‐sided).

## Results

### Clinicopathological features

**Figure 1:**
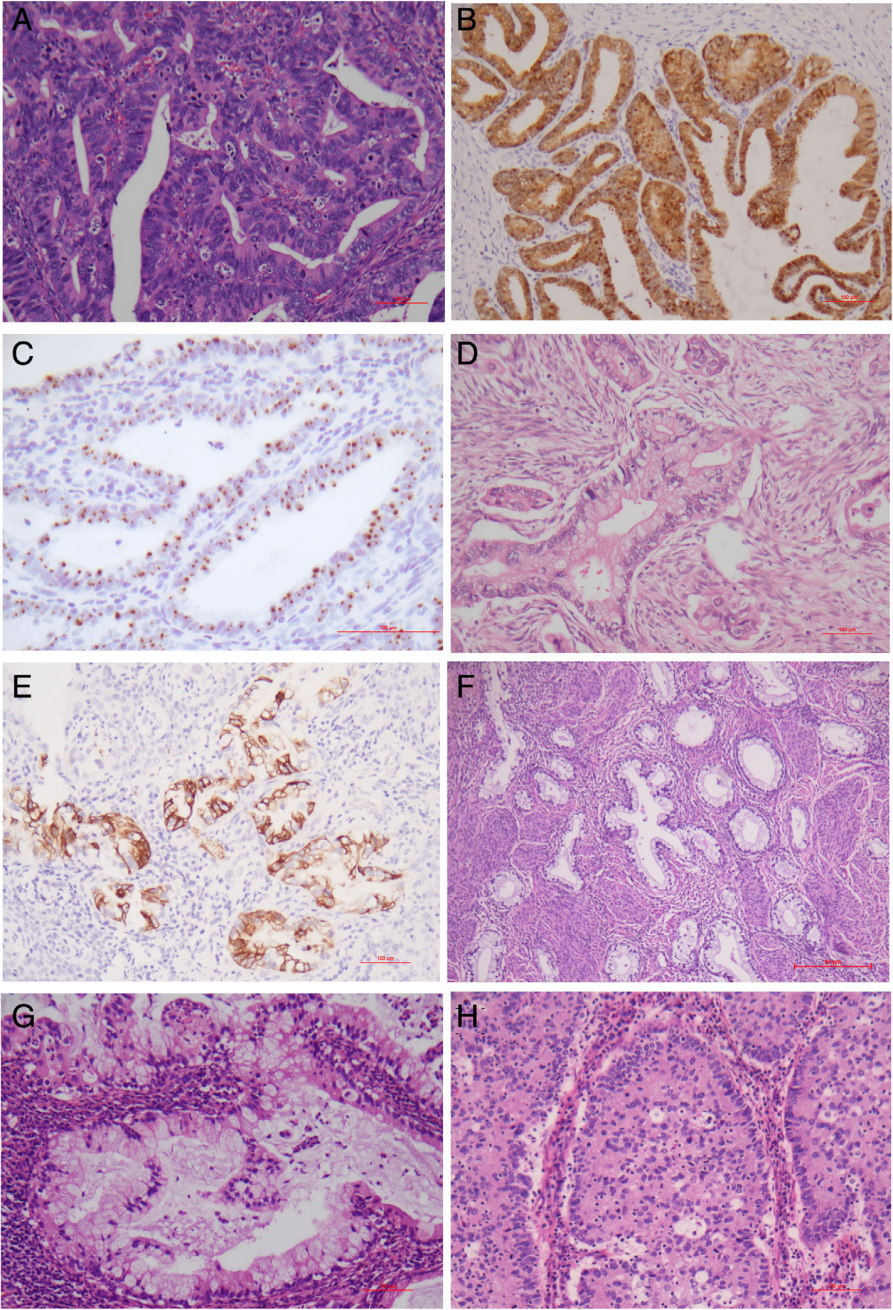
Histopathology of endocervical adenocarcinomas. (A–C) Endocervical adenocarcinoma, usual type. (A) Glandular and cribriform structures with appreciable intracytoplasmic mucin and frequent floating mitotic figures and apoptotic bodies. (B) Diffuse (block) p16 staining. (C) HPV mRNA signals. (D–F) Gastric‐type adenocarcinoma. (D) Invasive glands and tumor cells characterized by voluminous clear, foamy or eosinophilic cytoplasm, distinct cell borders and rounded, pale nuclei with distinct nucleoli. (E) MUC6 positivity. (F) GAC with well differentiated glands and bland cytology (minimal deviation adenocarcinoma). (G) mucinous adenocarcinoma NOS has >50% tumor cells with intracytoplasmic mucin in a background of adenocarcinoma, usual‐type. (H) Invasive stratified mucin‐producing carcinoma characterized by nests of stratified columnar cells with peripheral palisading and variable amounts of intracytoplasmic mucin. H&E: A, D, F–H; IHC: B, E; HPV mRNA ISH: C. Original magnifications: A, B, D‐F, H ×200; C ×400; G ×100.

The current 209 cases were composed of 159 (77%) HPV‐related and 50 (23%) non‐HPV‐related adenocarcinomas on the basis of the IECC (Table 1). The clinical and demographic information are given in supplementary material, Table S1. Representative histopathology is shown in Figure 1. Endocervical adenocarcinoma, usual‐type (111/209, 53.1%) (Figure 1A–C), and GAC (34/209, 16.3%) (Figure 1D–F) were the most common types of HPV‐related and non‐HPV‐related adenocarcinomas, respectively. In endocervical adenocarcinoma, usual‐type, the invasive pattern included Pattern A in 20 cases (18%), Pattern B in 24 cases (21.6%) and Pattern C in 67 cases (60.4%). Other relatively common histotypes included mucinous adenocarcinoma, not otherwise specified (NOS) (9.6%, 20/209) (Figure 1G) and invasive stratified mucin‐producing carcinoma (4.8%, 10/209) (Figure 1H). Lymphovascular invasion (LVI) and perineural involvement (PNI) were found in 73 and 11 patients, respectively. Both were more frequently present in GAC than in non‐GAC (LVI: 18/34, 52.9% versus 55/175, 31.4%, *p* < 0.05; PNI: 9/34, 26.5% versus 2/175, 1.1%, *p* < 0.001).

**Table 1:** Distribution of HER2 detection in cervical adenocarcinoma and histological subtypes.

Histological subtypes*	N	IHC 3+ (%)	FISH+ (%)
HPV‐related adenocarcinoma			
Usual type	111	12 (10.8%)	5 (4.5%)
Mucinous, NOS type	20	3(15%)	2(10%)
Stratified mucin‐producing type	10	0	0
Villoglandular type	7	0	0
Mucinous, intestinal type	1	0	0
Signet‐ring cell type	1	1 (100%)	1 (100%)
Non‐HPV‐related adenocarcinoma			
Gastric type	34	5 (14.7%)	5 (14.7%)
Serous type	4	0	0
Clear cell type	4	1 (25%)	0
Endometrioid type	3	0	0
Mesonephric type	1	0	0
Invasive adenocarcinoma, NOS	13	1 (7.7%)	0
Total	209	23 (11%)	13 (6.2%)

* The International Endocervical Adenocarcinoma Criteria and Classification (IECC) is applied.

Immunostaining demonstrated that most adenocarcinomas were negative for ER and PR except the endometrioid type. Diffuse (block) p16 staining (Figure 1B) or HPV signals (Figure 1C) were invariably found in HPV‐related adenocarcinomas with a frequency of 89.9% (143/159) or 91.2% (145/159), respectively. GAC was frequently positive for MUC6 (29/34, 85.3%) (Figure 1E). The Ki67 index ranged from 5 to 95% (median: 65%). It was much lower in GAC (37.3 ± 23.4%) than in the non‐GAC type (65.3 ± 16.8%) (*p* < 0.001). P53 overexpression was identified in 14 cases (14/188, 7.4%) and was more common in GAC (7/32, 21.9%) than in the non‐GAC type (4.5%, 7/156) (*p* = 0.003).

### HER2 status

**Figure 2:**
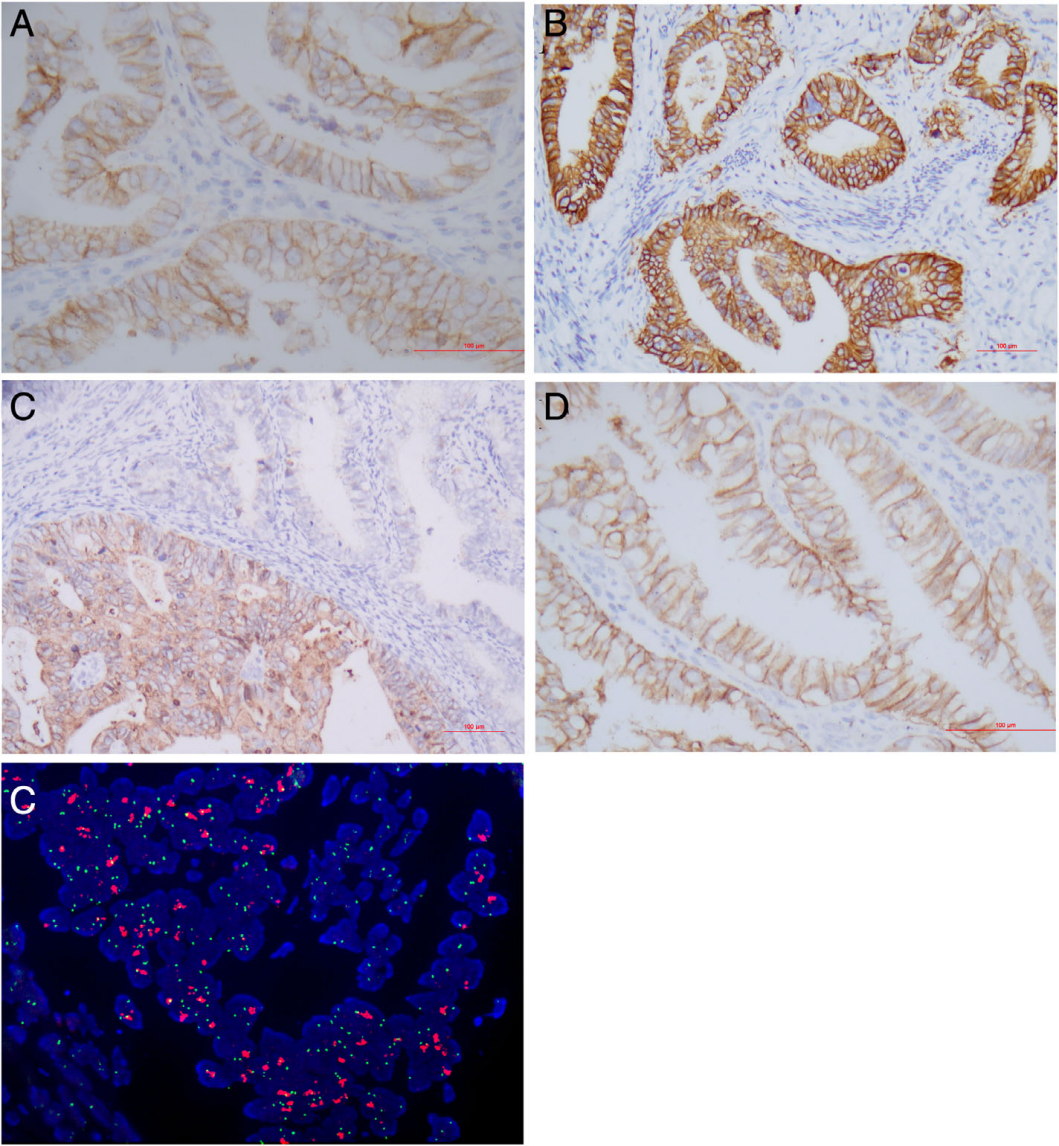
Detection of HER2 in cervical adenocarcinoma. (A, B) Representative areas of HER2 expression 2+, and 3+, respectively. (C) Heterogeneous HER2 staining. (D) Incomplete membranous (‘U’‐shaped) immunoreactivity. (E) *HER2* amplification by FISH. Immunohistochemistry: A–D; FISH: E. Original magnifications: A–C ×200; D, E ×400.

We observed HER2 expression in 123 (57.4%) adenocarcinomas which were composed of 1+ (negative) 62 cases; 2+ (equivocal) 38 cases (Figure 2A); and 3+ (positive) 23 cases (Figure 2B). The HER2 staining in cervical adenocarcinoma frequently showed an incomplete membrane (basolateral or ‘U’‐shaped) pattern (15/123, 12.2%) and intratumoral staining heterogeneity (21/123, 17.1%) (Figure 2C,D) resembling that in gastric and gastroesophageal adenocarcinoma rather than breast carcinoma. FISH analysis identified 13 cases (6.2%) with *HER2* amplification (Figure 2E). The concordance between FISH and IHC was 92.3% (193/209) as shown by 10/23 cases with IHC 3+; 3/38 cases with IHC 2+; 0/62 cases with IHC 1+, and 0/86 cases being FISH positive.

Among the major histotypes of endocervical adenocarcinomas, *HER2* amplification was relatively common in GAC (14.7%, 5/34) and mucinous adenocarcinoma, NOS (10%, 2/20) (Table 1). *HER2* amplification was more frequently associated with high tumor stage (FIGO III/IV), the GAC type, PNI and ovarian spread (*p* < 0.05) while IHC 3+ was more common in patients with LVI and ovarian involvement (*p* < 0.05) (Table 2).

**Table 2:** The clinicopathological features of cervical adenocarcinomas with HER2 positivity or gene amplification.

Clinicopathological features	IHC (%)		*P* value	FISH (%)		*P* value
	+^*^	−		+	−	
Age, mean ± SD (years)	51.2 ± 11.2	47.4 ± 9.7	0.080	48.3 ± 9.7	47.8 ± 10.0	0.863
FIGO stage			0.640			0.011
I and II	18	161		8	171	
III and IV	5	25		5	25	
HPV category^†^			0.793			0.310
HPV‐related	17	142		8	151	
Non‐HPV‐related	6	44		5	45	
Histotype			0.547			0.026
GAC	5	29		5	29	
Non‐GAC	18	157		8	167	
Patterns of invasion^‡^			0.122			0.583
A	0	20		0	20	
B and C	12	79		5	86	
Lymphovascular invasion			0.010			0.382
+	14	59		6	67	
−	9	127		7	129	
Perineural involvement			0.999			0.023
+	1	10		3	8	
−	22	176		10	188	
Ovarian involvement			0.031			<0.001
+	3	4		4	3	
−	20	182		9	193	

^*^ IHC 3+ was defined as HER2 positive.

^†^ Invasive adenocarcinomas, NOS were recategorized into non‐HPV (*n* = 4) and HPV‐related (*n* = 9) adenocarcinomas based on p16 staining and/or HPV RNA ISH detection.

^‡^ The pattern of invasion is only assessed in endocervical adenocarcinoma, usual type.

### Prognosis assessment

**Figure 3:**
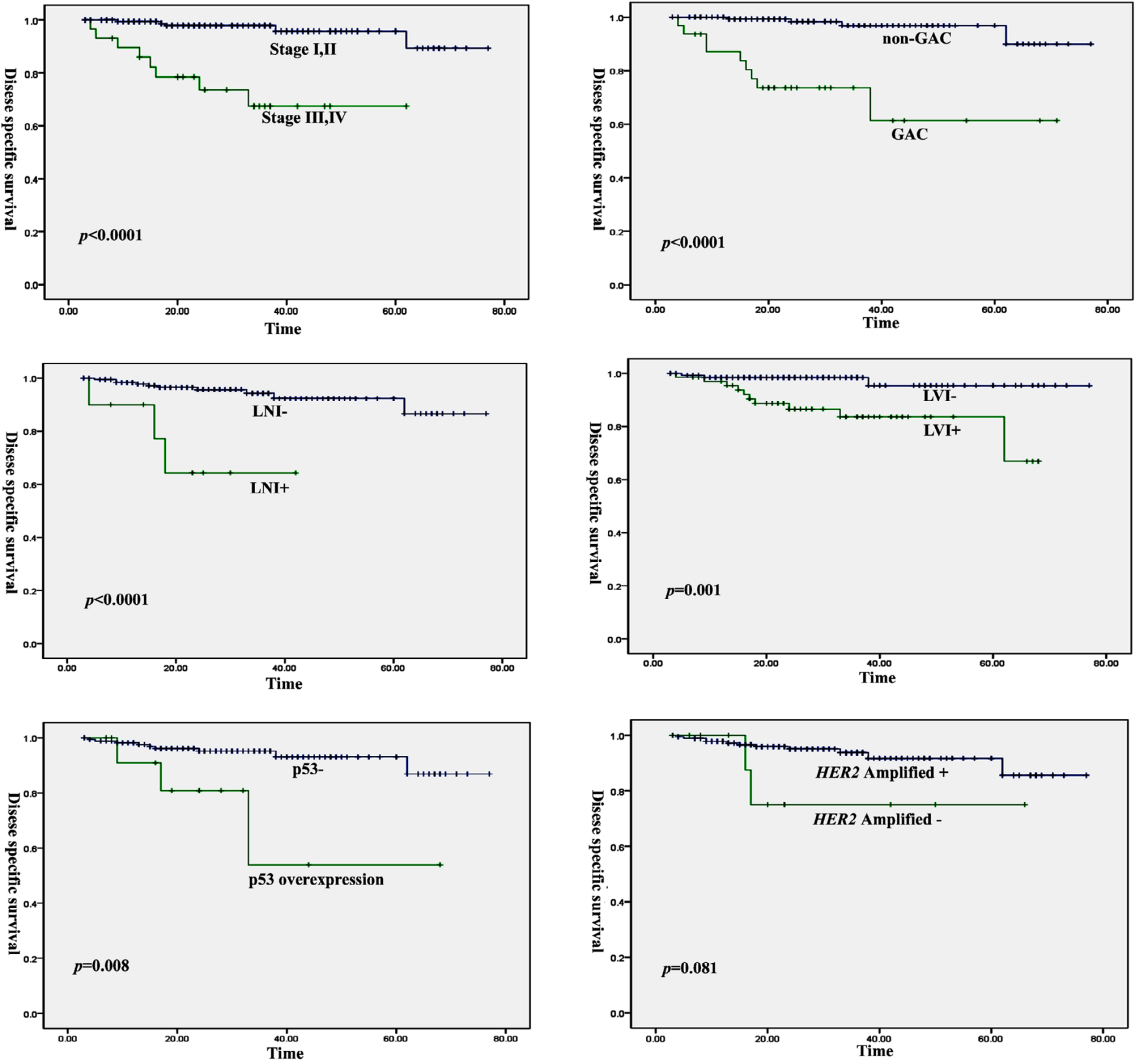
Kaplan–Meier curve for disease‐specific survival in cervical adenocarcinomas according to clinicopathological variables. FIGO stage III/IV, GAC, LNI, LVI and p53 overexpression are associated with poor prognosis and tumor recurrence in cervical adenocarcinomas (*p* < 0.05) while patients with *HER2* amplification show a marginal trend towards a worse overall survival.

Most patients (*n* = 197) underwent radical abdominal hysterectomy and bilateral salpingo‐oophorectomy and pelvic lymph‐node dissection. The FIGO stage included 163 cases at stage I, 18 at stage II, 26 at stage III and 4 at stage IV. GAC was more frequently found at FIGO stage III/IV (14/34, 41.2%) than non‐GAC (16/175, 9.1%) (*p* < 0.001). The patients were followed up for 3–77 months (median time: 25 months). A total of 24 patients recurred with a median time of 16.5 months (range: 2–61 months) post‐operatively. Thirteen patients died of disease (median time: 16 months; range: 4–62 months) and one died of other causes 17 months after surgery. In particular, we noted that 15 GAC patients recurred at a median time of 13 months (range 2–36 months) and 9 died of disease (median time: 15 months; range: 4–38 months). Survival analysis indicated that FIGO stage III/IV, GAC, or the non‐HPV‐related adenocarcinomas, LVI, PNI, and p53 overexpression were associated with poor disease‐specific survival in cervical adenocarcinomas (*p* < 0.05) (Figure 3). Moreover, these factors except p53 overexpression were also correlated with tumor recurrence (*p* < 0.05). *HER2* amplification or IHC3+ was not an indicator for adverse clinical outcomes in patients with endocervical adenocarcinomas (*p* > 0.05). However, the Kaplan–Meier plot showed a marginal trend towards a worse overall survival in patients with *HER2* amplification (Figure 3). Multivariate survival analysis including these characteristics indicated that GAC, FIGO stage and p53 overexpression were strong, independent markers for disease‐specific survival and tumor recurrence (Table 3).

**Table 3:** Multivariate survival analysis of patients with cervical adenocarcinomas according to Cox's proportional hazard regression method.

Characteristics*	Exp(*B*) (95% CI)	*P* value
Disease‐specific survival		
Histotypes (GAC versus non‐GAC)	7.09 (2.03–24.73)	0.002
FIGO stage (III, IV versus I, II)	3.81 (1.88–7.73)	<0.001
p53 overexpression (yes versus no)	1.68 (1.03–2.74)	0.038
Recurrence		
Histotypes (GAC versus non‐GAC)	10.36 (3.91–27.46)	<0.001
FIGO stage (III, IV versus I, II)	3.54 (2.16–5.78)	<0.001

* Non‐GAC, FIGO stage I/II and no p53 overexpression were regarded as the reference in the relevant group, respectively.

## Discussion

Numerous targeted therapies against aberrant molecules have been developed in the past three decades. Among these, the most successful might be the anti‐HER2 therapy, trastuzumab, which has been approved by FDA for the treatment of breast, gastric and gastroesophageal carcinomas with *HER2* gene amplification or protein overexpression. *HER2* gene amplification and/or protein overexpression have been discovered in some cancers in the female genital tract, such as uterine serous carcinoma and ovarian mucinous carcinoma [24, 25]. These findings suggest the potential value of trastuzumab therapy in these cancers.

HER2 protein overexpression has been reported in cervical carcinomas, mainly of squamous cell type, with a frequency varying from 1 to 21% using different methods and criteria [26]. However, few studies have so far explored HER2 expression in cervical adenocarcinomas. In an early study, c‐erb‐B2 (HER2) expression was detected in 34 of 44 (77%) cervical adenocarcinomas, with strong membrane staining in 11 of 44 (25%), which was associated with advanced tumor stage and poor prognosis [17]. More recently, Halle *et al* [18] reported HER2 overexpression in 22 of 70 (30%) cervical adenocarcinomas; here, there was no correlation with poor clinical outcome. Our study may represent the largest clinicopathological investigation on HER2 protein expression in cervical adenocarcinomas to date. We noted the frequent presence of incomplete membrane (basolateral or ‘U’‐shaped) staining and intra‐tumoral heterogeneity in cervical adenocarcinoma, leading to the adoption of the amended scoring system for gastric adenocarcinoma [21]. However, even with the modified criteria, the incidence of HER2 overexpression (IHC 3+) in our series was at the low end of what has been reported previously [17, 18]. The reasons for these conflicting results may be largely associated with certain technical issues, such as tissue fixation (duration of, and time between surgical removal and, fixation), tissue processing protocol, different antibodies to HER2, IHC staining protocols, and stringent criteria for the assessment of HER2 staining; and to a lesser extent, with the genetic background of cervical adenocarcinomas in Chinese populations.

To the best of our knowledge, *HER2* gene amplification has not been extensively analyzed in a large series of cervical adenocarcinoma with detailed histopathological assessment and clinical follow‐up. Our study showed that the frequency of *HER2* amplification by FISH was 6.2% in cervical adenocarcinomas. A chromogenic *in situ* hybridization analysis has shown that true *HER2* amplification was rare in invasive cervical squamous cell carcinomas (5/136 cases, 3.7%) using tissue microarrays [16]. Recent integrated genomic studies have indicated a greater tendency for the occurrence of *HER2* amplification and recurrent mutations in cervical adenocarcinoma than in squamous cell carcinoma [27, 28]. Moreover, Halle *et al* [18] found that *HER2* genomic alterations rather than protein overexpression were associated with poor disease‐specific survival in patients with cervical carcinoma despite the presence of a link between gene copy number and protein status. In the current study, exclusively on cervical adenocarcinomas, patients with *HER2* amplification showed a marginal trend towards a worse overall survival. Furthermore, *HER2* amplification was significantly associated with several potential indicators of poor prognosis, such as high FIGO stage, perineural invasion and ovarian involvement. Therefore, the patients with *HER2* amplification can be expected to benefit from the current trastuzumab treatment.

We observed discrepancies of potential clinical relevance between *HER2* gene amplification and protein expression, although there was a high concordance between the two tests. IHC 3+ neither detected all amplified cases nor invariably correlated with gene amplification, as reported previously in some cancer types including cervical cancer (mostly squamous cell carcinoma) and gastric cancer [18, 29]. According to the ASCO/CAP guideline [23], IHC 3+ is determined to be *HER2* (amplification) positive in breast cancer. However, our results clearly demonstrated that IHC 3+ alone did not confirm, and IHC 2+ did not exclude, the presence of *HER2* amplification in cervical adenocarcinoma. Therefore, we feel that, unlike breast cancer, tests for both IHC and FISH (for cases with IHC 2+ and 3+) are required for cervical adenocarcinoma patients in clinical trials of trastuzumab treatment, which is primarily aimed towards patients with *HER2* amplification. Likewise, such methodology has been recommended in clinical trials for trastuzumab treatment of gastric and gastroesophageal junction adenocarcinomas [14, 29]. The discrepancies between *HER2* amplification by FISH analysis and IHC results may suggest the presence of other factors activating *HER2* in cervical adenocarcinomas, such as recurrent somatic mutations, HPV integration, transcriptional upregulation of *HER2* without gene amplification, or polysomy leading to false positive IHC [24, 27, 28]. These tumor biology‐related molecular abnormalities indicate a potential for anti‐*HER2* therapy other than trastuzumab, such as pertuzumab and trastuzumab emtansine (T‐DM1), and pan‐*HER* inhibitors afatinib and neratinib [30, 31].

We systematically analyzed the distribution of *HER2* amplification and clinicopathological features across various subtypes of cervical adenocarcinomas using IECC, a new pathogenetic classification for invasive endocervical adenocarcinoma [5]. We found that non‐HPV‐related adenocarcinomas and GAC had a poor overall survival and shorter relapse time. These results add more evidence to support the prognostic value of IECC for endocervical adenocarcinoma and the aggressive nature of GAC [6, 7, 8, 9]. The distribution of *HER2* amplification among histotypes of cervical adenocarcinomas has not yet been fully investigated, although IHC studies demonstrated that endocervical adenocarcinomas of usual type had a HER2 positive frequency of 1.8% [5], which was much lower than in GAC [32]. It was estimated that 5–15% of cervical GAC may harbor *HER2* amplification [32]. In this study, we observed that the frequency of *HER2* amplification was 14.7% (5/34) in GAC, substantially higher than in other histotypes. Nevertheless, the precise level remains inconclusive because *HER2* amplification has been investigated in very few cases owing to its low incidence. Nakamura *et al* [32] detected only one GAC with *HER2* amplification from four cases with equivocal protein expression that were successfully analyzed by dual color *in situ* hybridization. Recently, Garg *et al* [33] identified *HER2* amplification by FISH in 2 of 12 cervical GACs. Taken together, the relatively high *HER2* amplification rate in cervical GAC, which was comparable to that reported in gastric cancers (12.2–20%) [14, 29, 34], may suggest the particular treatment implications for these patients on account of their poor clinical outcomes. However, we have to acknowledge that the prognostic and therapeutic significance of GAC should be further validated in clinical studies on more cases with long‐term follow‐up.

In this large case series, we demonstrated that p53 overexpression was an independent prognostic biomarker for cervical adenocarcinoma. Our findings are consistent with results from previous studies, which showed that p53 overexpression exerted an adverse impact on overall survival in cervical carcinomas including adenocarcinoma [18, 35]. In cervical carcinoma, it is well‐known that loss of p53 is caused by proteasomal degradation induced by HPV E6 protein, while overexpression is related to gene mutations [28, 36]. Severe dysregulation of cellular growth caused by *TP53* mutation may be the underlying mechanism for poorer survival in cervical cancer patients with p53 overexpression [18].

The limitations of our study include a small number of *HER2* amplified cases and GACs, the relatively short follow‐up time, and lack of well‐established evaluation criteria for HER2 expression in cervical adenocarcinoma. Further investigation of more *HER2*‐amplified cases and GAC with longer follow‐up time is required to develop more precise evaluation criteria for HER2 detection and to clarify the clinical significance of *HER2* amplification in cervical adenocarcinomas.

In conclusion, our large case series identified FIGO stage, GAC histotype and p53 overexpression as adverse prognostic features in cervical adenocarcinomas. *HER2* amplification was present in a subset of adenocarcinomas, and more commonly in GAC. Trastuzumab treatment is suggested as being beneficial for these patients although the prognostic value of gene amplification needs further validation. HER2 overexpression does not identify gene amplification status; therefore, our recommendation is to use FISH in IHC‐positive and equivocal cases.

## Author contributions statement

HS acquired, analysed and interpreted the data; and drafted the manuscript. YS acquired, analysed and interpreted the data; and participated in important discussions of the contents. WL designed the work and revised the manuscript. BL conceived and designed the work, revised the manuscript and approved the final version.

## Supporting information


**Table S1.** Clinical and demographic information for the IEEC subcategories
