# Supplementary material for: An analysis of HER2 amplification in cervical adenocarcinoma: correlation with clinical outcomes and the International Endocervical Adenocarcinoma Criteria and Classification
Source: J Pathol Clin Res. 2020 Oct 22;7(1):86–95. doi: 10.1002/cjp2.184 (PMC7737776; doi:10.1002/cjp2.184)
Supplement: Supplementary file 1 — Table S1. Clinical and demographic information for the IEEC subcategories [file CJP2-7-86-s001.docx]

**An analysis of HER2 amplification in cervical adenocarcinoma: correlation with clinical outcomes and the International Endocervical Adenocarcinoma Criteria and Classification**

H Shi *et al*. *J Pathol Clin Res* DOI: 10.1002/cjp2.184

**Table S1.** Clinical and demographic information for the IEEC subcategories

| **Main category** | **Morphological subtypes** | **N=209 (%)** | **Age (median, range; yr)** | **FIGO Stage (n)** |
| --- | --- | --- | --- | --- |
| HPV-related adenocarcinoma | Usual type | 111 (53.1%) | 46 (29-67) | I(98), II(6), III(7) |
|  | Villoglandular type | 7 (3.3%) | 39 (27-48) | I(7) |
|  | Mucinous NOS type | 20 (9.6%) | 44.5 (32-65) | I(16),II(2), III(2) |
|  | Stratified mucin-producing type | 10 (4.8%) | 45 (29-65) | I(6),II(1), III(3) |
|  | Signet-ring cell type | 1 (0.5%) | 48 | I(1) |
|  | Mucinous intestinal type | 1 (0.5%) | 50 | III(1) |
| Non-HPV related adenocarcinoma | Gastric-type | 34 (16.3%) | 48(33-77) | I(14), II(7), III(9), IV(4) |
|  | Endometrioid type | 3 (1.4%) | 54(45-57) | I(3) |
|  | Serous type | 4 (1.9%) | 59(37-64) | I(3),III(1) |
|  | Clear cell type | 4 (1.9%) | 61.5 (59-64) | I(2), II(1), III(1) |
|  | Mesonephric type | 1 (0.5%) | 49 | I(1) |
| Invasive adenocarcinoma, NOS |  | 13 (6.2%) | 48(27-68) | I(10), II(1), III(2) |

FIGO, International Federation of Gynecology & Obstetrics; IEEC, the International Endocervical Adenocarcinoma Criteria and Classification; yr, year(s); NOS, not otherwise specified.
